# Intra‐abdominal hemorrhage as a rare complication of endoscopic ultrasonography: A case report

**DOI:** 10.1002/deo2.80

**Published:** 2021-12-01

**Authors:** Kazunaga Ishigaki, Yousuke Nakai, Akiyuki Inokuma, Yukari Suzuki, Kensaku Noguchi, Sachiko Kanai, Tatsunori Suzuki, Tatsuya Sato, Ryunosuke Hakuta, Kei Saito, Tomotaka Saito, Naminatsu Takahara, Tsuyoshi Hamada, Suguru Mizuno, Hirofumi Kogure, Yudai Nakai, Mitsuhiro Fujishiro

**Affiliations:** ^1^ Department of Gastroenterology Graduate School of Medicine the University of Tokyo Tokyo Japan; ^2^ Department of Chemotherapy Graduate School of Medicine the University of Tokyo Tokyo Japan; ^3^ Department of Endoscopy and Endoscopic Surgery Graduate School of Medicine the University of Tokyo Tokyo Japan; ^4^ Department of Gastroenterology Japanese Red Cross Medical Center Tokyo Japan; ^5^ Department of Gastroenterology Kanto Central Hospital Tokyo Japan; ^6^ Department of Radiology Graduate School of Medicine the University of Tokyo Tokyo Japan

**Keywords:** complication, endoscopic ultrasonography, intra‐abdominal hemorrhage

## Abstract

Intra‐abdominal hemorrhage after endoscopic ultrasonography (EUS) is an uncommon complication, which can lead to potentially fatal outcomes. We describe a case of intra‐abdominal hemorrhage due to left gastric arterial bleeding after EUS. The patient developed severe epigastric pain 10 h after diagnostic EUS for pancreatic cysts. Contrast‐enhanced computed tomography revealed extravasation from the left gastric artery as well as a hematoma in the lesser omentum, which was confirmed by emergent angiography. Spontaneous hemostasis was obtained without embolization and the patient did not have further episodes of intra‐abdominal hemorrhage. Endoscopists should be aware of this rare but serious complication after endoscopic procedures.

## INTRODUCTION

Endoscopic ultrasonography (EUS) is often utilized for the evaluation of pancreato‐biliary diseases and gastrointestinal submucosal tumors. Although diagnostic EUS without tissue acquisition is safe and well‐tolerated, there is a small risk of complications such as bleeding and perforation.^1^, ^2^ While intraluminal bleeding and perforation are readily diagnosed during the procedures, intra‐abdominal hemorrhage is rare and may occur as a delayed event, which makes its diagnosis difficult. Herein, we describe a case of intra‐abdominal hemorrhage as a rare complication associated with diagnostic EUS.

## CASE REPORT

**FIGURE 1:**
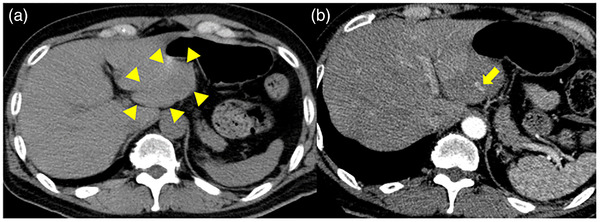
(a) Computed tomography (CT) without intravenous contrast media revealed the presence of hematoma around the stomach (arrowhead). (b) Dynamic CT showed contrast media extravasation from the branch of the left gastric artery (arrow) and hematoma in the lesser omentum

**FIGURE 2:**
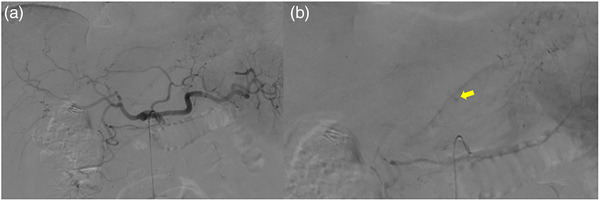
(a) Digital subtraction angiography from the celiac artery. (b) Contrast media extravasation from the branch of the left gastric artery (arrow) was confirmed

A 43‐year‐old man with a history of laparoscopic cholecystectomy underwent surveillance EUS for the first time to evaluate a 17‐mm intraductal papillary mucinous neoplasm without EUS‐guided fine needle aspiration. As a preoperative examination for laparoscopic cholecystectomy, he had undergone gastroduodenoscopy without any complications. The patient had no other comorbidity and did not take any medications including anti‐thrombotic agents. The procedure was completed uneventfully under moderate sedation with 5 mg *midazolam* and 15 mg *pentazocine*. Initially, this procedure was performed by a trainee, but the operator had changed to an expert during the procedure. He was discharged home without any symptoms. The patient developed severe epigastric pain after 10 h. On admission, the oral temperature was 36.0°C, blood pressure was 161/103 mmHg, pulse rate was 104/min, and respiratory rate was 16/min. The blood tests demonstrated only elevated levels of inflammatory markers, including a white blood cell count of 13,200/mm^3^ with a neutrophil left shift and a C‐reactive protein level of 0.37 mg/dl. Platelet count and blood coagulation test were within normal limits: platelet count, 306,000/μl; prothrombin time‐international normalized ratio (PT‐INR), 97.6%; activated partial thromboplastin time (APTT), 22.7 s. Physical examination revealed signs of peritoneal irritation. Computed tomography (CT) scan without contrast enhancement was performed to rule out perforation, which demonstrated no free air but revealed the presence of hematoma around the stomach and bloody ascites in the pelvis. Subsequent dynamic contrast‐enhanced CT (CECT) to identify the source of intra‐abdominal bleeding revealed hematoma in the lesser omentum and extravasation from the branch of the left gastric artery without evidence of aneurysm (Figure 1). Although the hemoglobin level did not drop significantly from 15.6 mg/dl prior to the procedure to 14.6 mg/dl on admission, angiography was performed due to severe abdominal pain and the evidence of extravasation on CT. The initial angiography from the celiac artery demonstrated extravasation from the branch of the left gastric artery, consistent with CT findings. However, extravasation disappeared on subsequent selective angiography from the left gastric artery, suggesting spontaneous hemostasis, and transcatheter arterial embolization (TAE) was not performed (Figure 2). The abdominal pain gradually improved and a follow‐up CT scan 2 days later showed no signs of extravasation and marked shrinkage of hematoma in the lesser omentum. The hemoglobin level did not drop any further and the patient was discharged without any symptoms.

## DISCUSSION

EUS is widely accepted as one of the diagnostic modalities in the field of gastroenterology including pancreatic cysts. Diagnostic EUS is safe but has a low but non‐negligible risk of complications such as bleeding, perforation, and infection due to its thick and rigid scope^1^, ^2^. Most bleeding by diagnostic EUS is mild, self‐limiting intraluminal bleeding and intra‐peritoneal hemorrhage has been reported only after EUS‐guided fine needle aspiration.^3^, ^4^

Intra‐abdominal hemorrhage is a rare but life‐threatening condition and may be either traumatic or non‐traumatic. The major cause of traumatic intra‐abdominal hemorrhage is blunt abdominal trauma, but iatrogenic hemoperitoneum caused by endoscopic procedures and surgical interventions can be encountered. Meanwhile, non‐traumatic intra‐abdominal hemorrhage known as spontaneous hemoperitoneum often arises from hepatic, splenic, or vascular pathology in patients with bleeding disorders.^5^ Clinical symptoms of intra‐abdominal hemorrhage consist of sudden abdominal pain and distention associated with an acute drop in hemoglobin levels. This condition necessitates urgent management including blood transfusion, TAE, and surgical intervention.

Intra‐abdominal hemorrhage after endoscopic procedures was reported after the colonoscopy due to the direct damage to the spleen during scope maneuvers around the splenic flexure.^6^, ^7^ Similarly, intra‐abdominal hemorrhage following upper gastrointestinal endoscopy has been less frequently reported,^8^, ^9^, ^10^ which may occur due to excessive tension on the gastric or duodenal wall by the scope manipulation. The scope manipulation of a thick and rigid EUS scope under the oblique endoscopic view can potentially increase the risk of intra‐abdominal hemorrhage. The presence of bleeding tendency and aneurysm can also be risk factors for bleeding; however, the patient did not have these risk factors. Platelet count and a blood coagulation test were within normal limits, and CECT images before cholecystectomy showed no aneurysm or arterial malformation, suggesting segmental arterial mediolysis, fibromuscular dysplasia, and polyarteritis nodosa. We speculate that the intraperitoneal hemorrhage, in this case, was due to an excessive extension of the gastric wall during the observation from the bulb of the duodenum in the long position. In addition, intra‐abdominal adhesions due to laparoscopic cholecystectomy might have contributed to the disruption of the left gastric artery. It is important to avoid excessive push manipulation during the observation, especially from the bulb of the duodenum, to prevent this rare complication.

In conclusion, we report a case of intra‐abdominal hemorrhage as a rare complication associated with diagnostic EUS. We should be aware of this rare but serious complication after endoscopic procedures to avoid the delay in its diagnosis and management.

## CONFLICT OF INTEREST

The authors declare that they have no conflict of interest.

## FUNDING INFORMATION

None.

## ETHICS STATEMENT

All procedures followed have been performed in accordance with the ethical standards laid down in the 1964 Declaration of Helsinki and its later amendments.

## HUMAN/ANIMAL RIGHTS

All procedures followed have been performed in accordance with the ethical standards laid down in the 1964 Declaration of Helsinki and its later amendments.

## INFORMED CONSENT

Informed consent was obtained from the patient for being published in this case report.

## References

[1] Jenssen C , Alvarez‐Sánchez MV , Napoléon B , Faiss S . Diagnostic endoscopic ultrasonography: Assessment of safety and prevention of complications. World J Gastroenterol 2012; 18: 4659–76. 23002335 10.3748/wjg.v18.i34.4659 PMC3442204

[2] Lakhtakia S . Complications of diagnostic and therapeutic endoscopic ultrasound. Best Pract Res Clin Gastroenterol 2016; 30: 807–23. 27931638 10.1016/j.bpg.2016.10.008

[3] Carrara S , Arcidiacono PG , Giussani A , Testoni PA . Acute hemorrhage with retroperitoneal hematoma after endoscopic ultrasound‐guided fine‐needle aspiration of an intraductal papillary mucinous neoplasm of the pancreas. Am J Gastroenterol 2009; 104: 1610–1. 19491887 10.1038/ajg.2009.132

[4] Lew SQ , Khan AA , Rieders B , Agrawal ST . Haemoperitoneum after an endoscopic ultrasound‐guided fine‐needle aspiration (EUS‐FNA) of a pancreatic lesion in a peritoneal dialysis patient. BMJ Case Rep 2020; 13: e236573. 10.1136/bcr-2020-236573 PMC7643458 33148597

[5] Lucey BC , Varghese JC , Soto JA . Spontaneous hemoperitoneum: Causes and significance. Curr Probl Diag Radiol 2005; 34: 182–95. 10.1067/j.cpradiol.2005.06.001 16129236

[6] Janes SE , Cowan IA , Dijkstra B . A life threatening complication after colonoscopy. BMJ 2005; 330: 889–90. 15831876 10.1136/bmj.330.7496.889 PMC556163

[7] Tagg W , Woods S , Razdan R , Gagliardi J , Steenbergen P . Hemoperitoneum after colonoscopy. Endoscopy 2008; 40(Suppl 2): E136–7. 18633865 10.1055/s-2007-995715

[8] Dehn TC , Lee EC . Intraperitoneal hemorrhage following fiberoptic gastroscopy. Gastrointest Endosc 1985; 31: 350. 10.1016/s0016-5107(85)72227-4 3876256

[9] Pricolo R , Cipolletta L . Intraperitoneal hemorrhage following upper gastrointestinal endoscopy. Gastrointest Endosc 1987; 33: 53–4. 10.1016/s0016-5107(87)71497-7 3557042

[10] Ueda M , Yamaguchi H , Kagawa Y *et al*. [Intra‐abdominal bleeding caused after esophagogastroduodenoscopy: A case report]. Nihon Shokakibyo Gakkai Zasshi 2020; 117: 985–91. 33177261 10.11405/nisshoshi.117.985

